# Supplementary material for: National Near Real-Time Vaccine Effectiveness Against COVID-19 Severe Outcomes Using the Screening Method Among Older Adults Aged ≥50 Years in Canada
Source: Vaccines (Basel). 2025 Dec 24;14(1):26. doi: 10.3390/vaccines14010026 (PMC12846497; doi:10.3390/vaccines14010026)
Supplement: Supplementary file 1 [file vaccines-14-00026-s001.zip › Figure S3.pptx]

## Slide 1
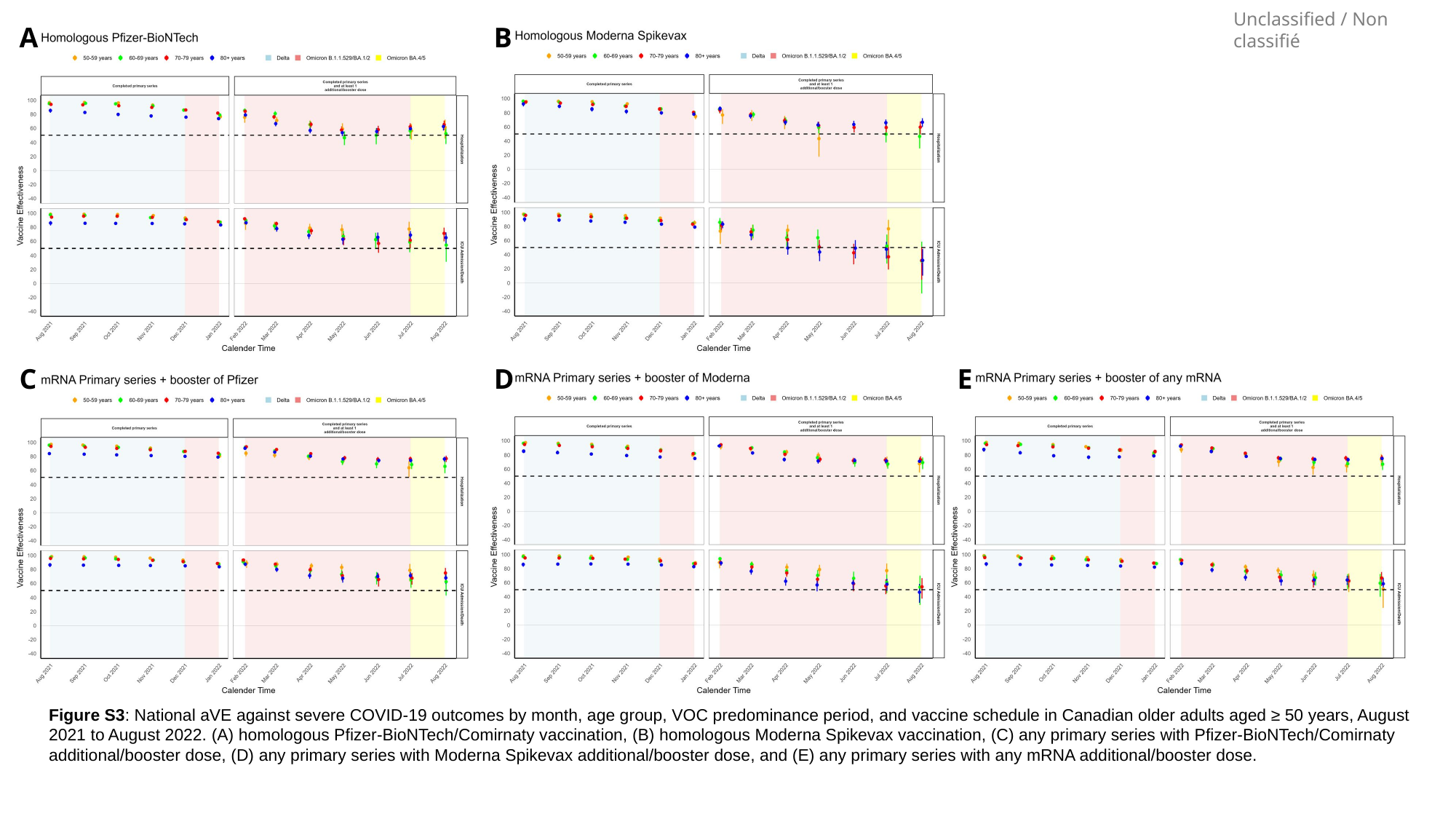

A
B
C
D
E
Figure S3: National aVE against severe COVID-19 outcomes by month, age group, VOC predominance period, and vaccine schedule in Canadian older adults aged ≥ 50 years, August 2021 to August 2022. (A) homologous Pfizer-BioNTech/Comirnaty vaccination, (B) homologous Moderna Spikevax vaccination, (C) any primary series with Pfizer-BioNTech/Comirnaty additional/booster dose, (D) any primary series with Moderna Spikevax additional/booster dose, and (E) any primary series with any mRNA additional/booster dose.
