# Supplementary material for: National Near Real-Time Vaccine Effectiveness Against COVID-19 Severe Outcomes Using the Screening Method Among Older Adults Aged ≥50 Years in Canada
Source: Vaccines (Basel). 2025 Dec 24;14(1):26. doi: 10.3390/vaccines14010026 (PMC12846497; doi:10.3390/vaccines14010026)
Supplement: Supplementary file 1 [file vaccines-14-00026-s001.zip › vaccines-4044031-supplementary.pdf]

vaccines

MDPI

Supplementary Information

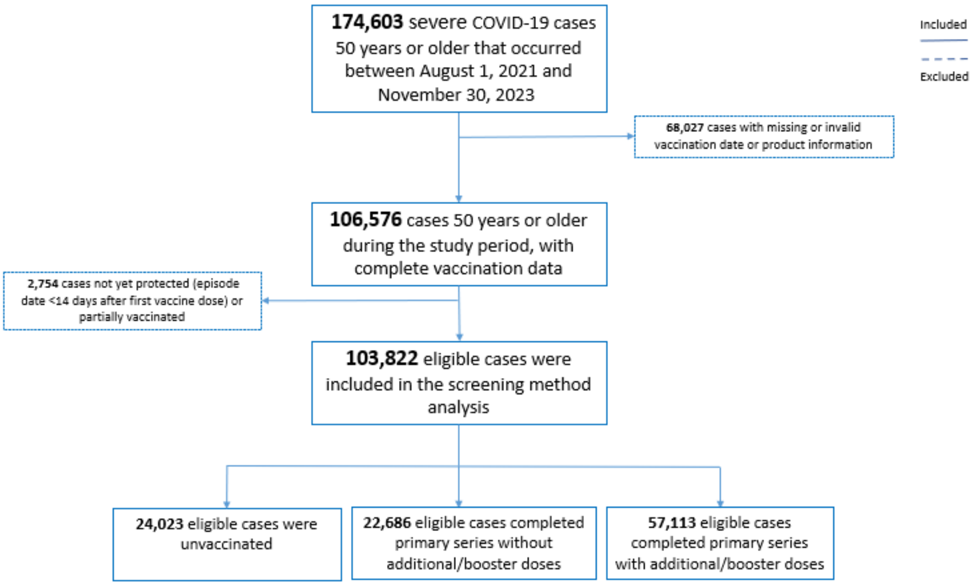

Figure S1. Participant flowchart for severe COVID-19 cases aged ≥ 50 years and the exclusion criteria applied for the screening method in Canada, August 1, 2021 to November 30, 2023.

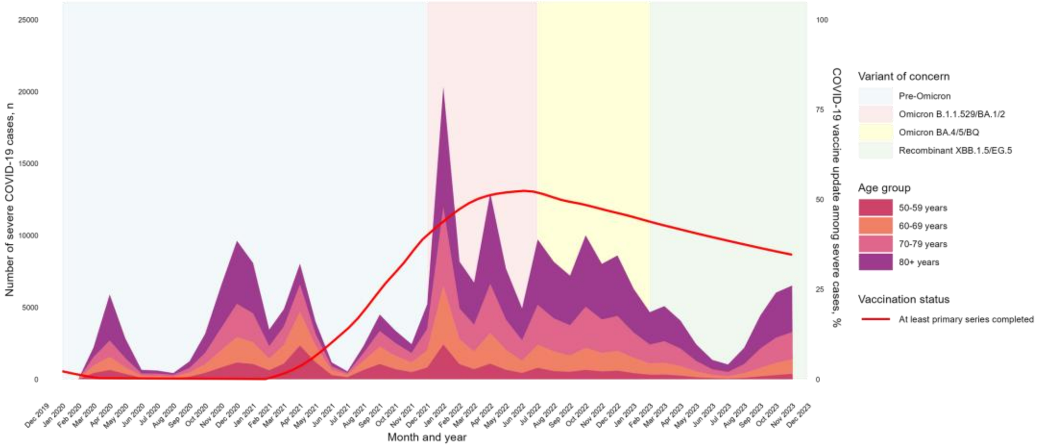

Figure S2. Epidemic curve of severe COVID-19 cases by age group and VOC, Canada, December 2019 to November 2023.

Table S1. COVID-19 vaccine uptake and schedule among severe COVID-19 cases in older adults aged ≥ 50 years, August 1, 2021 to November 30, 2023.

Characteristic	Primary Series Completed (n=22,686)		Primary Series Completed with One Additional/Booster Dose (n=33,926)		Primary Series Completed with Two or More Additional/Booster Doses (n=23,187)	
	n	%	*n*	%	*n*	%
**Homologous and heterologous schedule among primary series (n=22,686)**						
Two doses-Homologous Pfizer-BioNTech/Comirnaty (BNT162b2) [PF/PF]	14,606	64.4	–	–	–	–
2 dose–Homologous Moderna Spikevax (mRNA–1273) [MD/MD]	4959	21.9	–	–	–	–

**Characteristic**	**Primary Series Completed (n=22,686)**		**Primary Series Completed with One Additional/Booster Dose (n=33,926)**		**Primary Series Completed with Two or More Additional/Booster Doses (n=23,187)**	
	n	**%**	***n***	**%**	***n***	**%**
Two dose–Received other vaccine in series	20	0.1	–	–	–	–
Two doses–Heterologous or Mixed series of mRNA vaccines [PF/MD or MD/PF]	2056	9.1	–	–	–	–
Two doses–Homologous Astra-Zeneca/COVISHIELD (ChAdOx1 nCoV–19) [AZ/AZ]	418	1.8	–	–	–	–
Two doses– Heterologous Astra-Zeneca Vaxzevria/COVISHIELD with an mRNA vaccine [AZ/PF or AZ/MD]	627	2.8	–	–	–	–
**Homologous and heterologous schedule among primary series with one additional/Booster dose (n=33,926)**						
Three doses–Homologous Pfizer–BioNTech/Comirnaty (BNT162b2) [PF/PF/PF]	–	–	18,382	54.2	–	–
3 doses–Homologous Moderna Spikevax (mRNA–1273) [MD/MD/MD]	–	–	5663	16.7	–	–
Three doses–Heterologous mRNA primary series followed by one or more additional/booster doses of Pfizer–BioNTech/Comirnaty (BNT162b2) [mRNA/mRNA/PF]	–	–	1681	5	–	–
Three doses–Heterologous mRNA primary series followed by one or more additional/booster doses of Moderna Spikevax (mRNA–1273) [mRNA/mRNA/MD]	–	–	6857	20.2	–	–
Three doses–Heterologous Astra-Zenca primary series followed by any mRNA additional/booster doses [AZ/AZ/mRNA]	–	–	415	1.2	–	–
Three doses–Other vaccine series	–	–	928	2.7	–	–
**Homologous and heterologous schedule among primary series with two or more additional/Booster doses (n=23,187)**						
≥4 doses-Homologous Pfizer-BioNTech/Comirnaty (BNT162b2) [PF/PF/PF/PF]	–	–	–	–	9596	41.4
≥4 doses -Homologous Moderna Spikevax (mRNA-1273) [MD/MD/MD/MD]	–	–	–	–	3354	14.5
≥4 doses - Heterologous mRNA primary series followed by two or more additional/booster doses of Pfizer-BioNTech/Comirnaty (BNT162b2) [mRNA/mRNA/mRNA/PF]	–	–	–	–	2395	10.3
≥4 doses -Heterologous mRNA primary series followed by two or more additional/booster doses of Moderna Spikevax (mRNA-1273) [mRNA/mRNA/mRNA/MD]	–	–	–	–	5396	23.3
≥4 doses -Heterologous mRNA primary series followed by any mRNA additional/booster doses [mRNA/mRNA/mRNA/mRNA]	–	–	–	–	1815	7.8
≥4 doses -Heterologous Astra-Zenca primary series followed by	–	–	–	–	216	0.9

Characteristic	Primary Series Completed (n=22,686)		Primary Series Completed with One Additional/Booster Dose (n=33,926)		Primary Series Completed with Two or More Additional/Booster Doses (n=23,187)	
	n	%	*n*	%	*n*	%
any mRNA additional/booster doses [AZ/AZ/mRNA/mRNA]						
≥4 doses-Other vaccine series	–	–	–	–	412	1.8
≥3 doses - Heterologous Janssen primary series followed by any mRNA additional/booster doses [JJ/mRNA/mRNA]	–	–	–	–	3	0
**Most recent dose product received among primary series with or without additional/Booster doses (n=79,799)**						
Pfizer-BioNTech/Comirnaty ancestral monovalent	4681	20.6	6434	19	767	3.3
Pfizer-BioNTech/Comirnaty bivalent (BA.4/BA.5)	8	0	65	0.2	2513	10.8
Pfizer-BioNTech/Comirnaty (unspecified MV or BV)	10,658	47.0	13,091	38.6	3503	15.1
Pfizer-BioNTech/Comirnaty monovalent XBB.1.5	–	–	2	0	40	0.2
Moderna Spikevax ancestral monovalent	2546	11.2	6444	19	219	0.9
Moderna Spikevax bivalent (BA.1 or BA.4/BA.5)	14	0.1	117	0.3	3217	13.9
Moderna Spikevax (unspecified MV or BV)	4336	19.1	6219	18.3	2850	12.3
Moderna Spikevax monovalent XBB.1.5	1	0	1	0	83	0.4
Other COVID-19 vaccines [AstraZeneca Vaxzevria/COVISHIELD/ Janssen Jcovden / Novavax Nuvaxovid / Medicago Covifenz]	442	1.9	21	0.1	1	0
Vaccine product unknown	–	–	1,532	4.5	9,994	43.1
**Months since last dose among primary series with two or more additional/Booster doses (n=79,799)**						
14 days to 3 months	1281	5.6	5668	16.7	3226	13.9
3 to 6 months	5178	22.8	11,397	33.6	4851	20.9
6 to 9 months	7813	34.4	6901	20.3	2535	10.9
9 to 12 months	2994	13.2	4165	12.3	1478	6.4
Over 12 months	5420	23.9	4263	12.6	1103	4.8
Vaccine time unknown	–	–	1532	4.5	9994	43.1
**Time-interval between doses by last two products received among primary series with two or more additional/Booster doses (n=79,799)**						
0 to 27 days	1487	6.6	8	0.0	–	–
28 to 55 days	7171	31.6	66	0.2	3	0.0
56 to 83 days	7614	33.6	260	0.8	62	0.3
84 to 111 days	4205	18.5	800	2.4	1172	5.1
112 to 167 days	1746	7.7	6053	17.8	5253	22.7
≥168 days	463	2.0	25,207	74.3	6703	28.9
Vaccine time unknown	–	–	1532	4.5	9994	43.1

Abbreviations: Values represent the total count and proportion of cases by descriptive variable and vaccine status. **mRNA** = messenger ribonucleic acid**;** **PF**= Pfizer-BioNTech/Comirnaty; **MD**=Moderna Spikevax. **BV** = bivalent; **MV** = monovalent; **NA**=not applicable; **SMD** = standardized mean or proportion difference. **Other COVID-19 vaccines**: AstraZeneca Vaxzevria /COVISHIELD [**AZ**]; Janssen Jcovden [**JJ**]**;** Novavax Nuvaxovid; Medicago Covifenz.

 https://doi.org/10.3390/vaccines14010026

Table S2. Canadian aVE results among older adults using test-negative design (TND) by outcome, province/territory, vaccine status, and age group.

Outcome	Province/Territory	Vaccine Status	Age Group	VE Estimate (%)[ђ]	LO 95% CI (%)[ђ]	HI 95% CI (%)[ђ]	Reference
**Delta (August 2021 - November 2021)**							
**Hospitalization or death**	Ontario	Primary series completed	≥70 years	95.0 to 97.0	93.0 to 96.0	96.0 to 97.0	Chung et al., 2022
	Ontario	Primary series completed	≥60 years	96.0 to 96.0	70.0 to 93.0	98.0 to 99.0	Nasreen et al., 2022
**Omicron B.1.1.529/BA.1/BA.2 (December 2021 - June 2022)**							
**Hospitalization or death**	Ontario	Primary series completed	50-59 years	83.0 to 83.0	74.0 to 76.0	88.0 to 89.0	Grewal et al., 2023a
			60-69 years	79.0	71.0	86.0	
			70-79 years	80.0	72.0	86.0	
			≥80 years	72.0	62.0	79.0	
		Primary series and one additional/booster dose	50-59 years	96.0 to 98.0	93.0 to 93.0	97.0 to 99.0	
			60-69 years	90.0 to 95.0	79.0 to 93.0	95.0 to 96.0	
			70-79 years	91.0 to 96.0	85.0 to 96.0	95.0 to 97.0	
			≥80 years	87.0 to 92.0	80.0 to 90.0	91.0 to 93.0	
		Primary series and two additional/booster doses	50-59 years	97.0	77.0	100.0	
			60-69 years	95.0 to 96.0	77.0 to 93.0	98.0 to 99.0	
			70-79 years	93.0 to 96.0	87.0 to 94.0	96.0 to 97.0	
			≥80 years	92.0 to 95.0	78.0 to 92.0	95.0 to 97.0	
		Primary series completed	LTCF residents ≥60 years	52.0	33.0	65.0	Grewal et al., 2022
		Primary series and one additional/booster l dose		77.0 to 81.0	67.0 to 74.0	82.0 to 89.0	
		Primary series and two additional/booster doses		83.0 to 88.0	54.0 to 82.0	90.0 to 94.0	
**Hospitalization**	Quebec	Primary series completed	≥60 years	60.0 to 99.0	−13.0 to 96.0	67.0 to 100.0	Carazo et al., 2023
		Primary series and one additional/booster dose		66.0 to 93.0	48.0 to 92.0	78.0 to 94.0	
		Primary series and two additional/booster doses		82.0 to 96.0	69.0 to 93.0	86.0 to 98.0	
**Hospitalization or death**	Ontario	Primary series completed	≥50 years	80.0 to 80.0	77.0 to 78.0	82.0 to 83.0	Lee et al., 2023
		Primary series and one additional/booster dose		94.0 to 94.0	93.0 to 94.0	95.0 to 95.0	
		Primary series and two additional/booster doses		97.0	96.0	98.0	
**Omicron BA.4/BA.5/BQ (July 2022 - January 2023)**							
**Hospitalization or death**	Ontario	Primary series and one additional/booster dose	50-59 years	75.0 to 77.0	50.0 to 60.0	84.0 to 89.0	Grewal et al., 2023a
			60-69 years	69.0 to 94.0	53.0 to 60.0	78.0 to 99.0	
			70-79 years	59.0 to 86.0	37.0 to 44.0	70.0 to 97.0	
			≥80 years	52.0 to 76.0	27.0 to 36.0	64.0 to 92.0	
		Primary series and two additional/booster doses	50-59 years	95.0	82.0	98.0	
			60-69 years	81.0 to 88.0	70.0 to 78.0	88.0 to 94.0	
			70-79 years	85.0 to 86.0	77.0 to 80.0	90.0 to 90.0	
			≥80 years	80.0 to 86.0	72.0 to 79.0	85.0 to 90.0	
		Primary series and two additional/booster doses	≥50 years	76.0 to 91.0	66.0 to 88.0	83.0 to 93.0	Grewal et al., 2023b
**Hospitalization**	Quebec	Primary series completed	≥60 years	40.0 to 92.0	−95.0 to 85.0	49.0 to 95.0	Carazo et al., 2023
		Primary series and one additional/booster dose		56.0 to 82.0	45.0 to 68.0	64.0 to 90.0	
		Primary series and two additional/booster doses		57.0 to 80.0	−9.0 to 79.0	72.0 to 94.0	
**Hospitalization or death**	Ontario	Primary series completed	≥50 years	43.0 to 49.0	19.0 to 41.0	56.0 to 60.0	Lee et al., 2023
		Primary series and one additional/booster dose		67.0 to 69.0	62.0 to 63.0	71.0 to 74.0	

Outcome	Province/Territory	Vaccine Status	Age Group	VE Estimate (%)ᵇ	LO 95% CI (%)ᵇ	HI 95% CI (%)ᵇ	Reference
		Primary series and two additional/booster doses		78.0 to 95.0	74.0 to 93.0	82.0 to 97.0	
		**Recombinant XBB.1/EG.5 (February 2023 - November 2023)**					
**Hospitalization or death**	Ontario	Primary series completed	≥50 years	25.0 to 31.0	−61.0 to 17.0	43.0 to 65.0	Lee et al., 2023
		Primary series and one additional/booster dose		56.0 to 66.0	36.0 to 52.0	53.0 to 76.0	
		Primary series and two additional/booster doses		47.0 to 76.0	35.0 to 71.0	56.0 to 79.0	

SM: screening method; **VE**: vaccine effectiveness; **LO 95% CI**: lower 95% confidence interval; **HI 95% CI**: upper 95% confidence interval; **LTCF**: Long-term care facility ᵇ VE estimate ranges
